# Consensus-Based Interventions for Cervical and Lumbar Facet Joint Pain and Sacroiliac Joint Disorders: A Concise Summary of International Practice Guidelines

**DOI:** 10.7759/cureus.98977

**Published:** 2025-12-11

**Authors:** Roy Sebastian, Seung J Lee, Kanchana Gattu, Thelma Wright

**Affiliations:** 1 Anesthesiology, University of Maryland School of Medicine, Baltimore, USA; 2 Anesthesiology, University of Maryland Medical Center, Baltimore, USA

**Keywords:** aspn guidelines, cervical facet joint interventions, diagnostic blocks, fluoroscopic guidance, interventional pain medicine, lumbar facet joint interventions, radiofrequency ablation, sacroiliac joint injections

## Abstract

Cervical and lumbar facet joint interventions and sacroiliac joint injections are some of the most frequent interventions done in interventional pain medicine. Various detailed guidelines have been developed by international, multispecialty societies and panels, including the American Society of Pain and Neuroscience (ASPN), to guide clinical decision-making. However, these extensive documents are often hard to apply within the time limits of clinical practice. We compared and summarized three international working groups' and ASPN's published consensus guidelines' recommendations. The summary focuses on diagnostic criteria, procedure guidelines, and follow-up. All three guidelines emphasize the use of controlled diagnostic blocks, fluoroscopic or CT guidance, and consideration of radiofrequency ablation following diagnostic confirmation. Functional improvement and ≥50-80% pain relief thresholds are significant criteria for procedural progression. These brief summaries give pain physicians an easily accessible clinical guide to evidence-based consensus on spinal interventions. Full credit is given to the original guideline publications for their comprehensive work.

## Introduction and background

Spinal pain is highly prevalent and a leading cause of disability worldwide. Facet joints are synovial joints that enable spinal motion, whereas sacroiliac (SI) joints are partially synovial and partially fibrous, providing pelvic stability. Facet and SI joints are identified as common pain generators, and chronic spinal pain originating from these joints poses significant challenges in practice due to their multifactorial nature and impact on the quality of life of patients [1]. Hence, diagnosis is paramount for proper management. Diagnostic blocks identify the joint responsible for the symptoms, while injections or radiofrequency ablation (RFA) can provide therapeutic interventions to alleviate pain from the joint. The need for evidence-informed, technically sound interventional pain management has never been more crucial with the current trend toward value-based medicine [2]. Several professional organizations, such as the American Society of Pain and Neuroscience (ASPN) and other international guideline bodies, have agreed upon recommendations for the uniformity of such procedures, although consensus varies among guidelines [3]. This review summarizes the most recent guidelines from the PubMed database and ASPN repository. It provides recommendations to help clinicians make appropriate, timely, evidence-based decisions in the management of spinal pain.

While these guidelines are helpful, their clinical utility is lessened by technical vocabulary, specificity, and considerable depth, factors that lean toward restricting their usability to time-constrained clinicians. To address this failing, an accessible, practice-oriented distillation is needed to extract the chief recommendations from such authoritative sources without compromising their scientific acceptability. By issuing clear grades of recommendation and utilitarian guidance, such a tool can make clinical decision-making more streamlined, promote consistency in care, and ultimately lead to improved results in patients with chronic spinal pain.

## Review

Methods

This review aimed to summarize the most current guidelines from recognized societies. Specifically, this is a qualitative, narrative review rather than a systematic review, with the primary aim of summarizing and comparing current guidelines from recognized professional societies. As such, formal systematic review procedures, such as the Preferred Reporting Items for Systematic Reviews and Meta-Analyses (PRISMA) or quantitative meta-analysis, were not applied. The focus was on providing a concise synthesis of guideline recommendations to support evidence-based clinical decision-making.

Inclusion criteria included guidelines published by recognized professional societies, available in English, and representing the most recent versions with recommendations on diagnostic and procedural management.

Exclusion criteria included drafts, older versions, consensus statements, and expert reviews.

Grades of recommendation

The grades of recommendation table provides a uniform way to rate the strength of clinical recommendations relative to the quality of evidence. It aids in decision-making at the bedside by categorizing recommendations as strong or weak, depending on study design, consistency, and directness of the evidence. The system ensures transparency and consistency in clinical guidelines. One of the most widely utilized systems is the GRADE (Grading of Recommendations Assessment, Development and Evaluation) system, which is favored by most global health organizations [4]. The recommendations outlined in this summary are supported by the grading system presented in Table 1, which categorizes the grades of recommendation based on the strength and certainty of evidence used to guide clinical decision-making.

**Table 1:** Grades of practice recommendations.

Grade	Descriptor	Qualifying evidence	Implications for practice
A	Strong recommendation	Level I evidence or consistent findings from multiple studies of levels II, III, or IV	Clinicians should follow a strong recommendation unless a clear and compelling rationale for an alternative approach is present
B	Recommendation	Levels II, III, or IV evidence and findings are generally consistent	Generally, clinicians should follow a recommendation, but should remain alert to new information and sensitive to patient preferences
C	Option	Levels II, III, or IV evidence, but findings are inconsistent	Clinicians should be flexible in their decision-making regarding appropriate practice, although they may set bounds on alternatives; patient preference should have a substantial influencing role
D	Option	Level V evidence: little or no systematic empirical evidence	Clinicians should consider all options in their decision-making and be alert to new published evidence that clarifies the balance of benefit versus harm; patient preference should have a substantial influencing role
I	Not recommended	Insufficient evidence to make a recommendation	The balance between the potential benefits and potential harms is not clear

Levels of certainty regarding net benefit

The U.S. Preventive Services Task Force decides the certainty of the net benefit of preventive services by grading evidence for each key question, considering how well it is applicable to real-world scenarios. While randomized controlled trials (RCTs) are often used, their findings may not be generalizable due to selective populations and controlled settings, so the Task Force looks at the applicability of such evidence to more general clinical practice. Harms are also evaluated separately and on the same level as benefits, often from observational evidence since RCTs may not capture real-world risks like higher complication rates in community settings. The Task Force synthesizes all the evidence within key questions to determine the overall certainty of net benefit, which is high, moderate, or low based on the quality, consistency, and applicability of the evidence [5]. Table 2 exhibits the U.S. Preventive Services Task Force levels of certainty regarding net benefit provides the framework used to assess the overall strength and applicability of the evidence for their recommendations.

**Table 2:** U.S. Preventive Services Task Force levels of certainty regarding net benefit.

Level of certainty	Description
High	The available evidence usually includes consistent results from a multitude of well-designed, well-conducted studies in representative primary care populations. These studies assess the effects of the preventive service on the desired health outcomes. Because of the precision of findings, this conclusion is therefore unlikely to be strongly affected by the results of future studies. These recommendations are often based on direct evidence from clinical trials of screening or behavioral interventions. High-quality trials designed as “pragmatic” or “effectiveness” trials are often of greater value in understanding external validity.
Moderate	The available evidence is sufficient to determine the effects of the preventive service on targeted health outcomes, but confidence in the estimate is constrained by factors such as: • The number, size, or quality of individual studies in the evidence pool. • Some heterogeneity of outcome findings or intervention models across the body of studies. • Mild to moderate limitations in the generalizability of findings to routine primary care practice. As more information becomes available, the magnitude or direction of the observed effect could change, and this change may be large enough to alter the conclusion.
Low	The available evidence is insufficient to assess effects on health outcomes. Evidence is insufficient because of: • The very limited number or size of studies. • Inconsistency of direction or magnitude of findings across the body of evidence. • Critical gaps in the chain of evidence. • Findings are not generalizable to routine primary care practice. • A lack of information on prespecified health outcomes. • Lack of coherence across the linkages in the chain of evidence. More information may allow an estimation of effects on health outcomes.

Cervical facet joint interventions

Over the last two decades, there has been an exponential growth in the use of cervical spine joint interventions such as joint injections, nerve blocks, and RFA in the treatment of chronic neck pain, and the analysis of procedural data supports it. For example, one study of US Medicare data between 2000 and 2009 found a ~310% overall increase in facet joint interventions, while from 2009 to 2018, the overall use still rose, albeit more slowly, by another 18.8% per 100,000 Fee-For-Service Medicare enrollees [6]. More recently, from 2010 through 2019, procedures for cervical facet-joint injection increased approximately 2% a year, whereas cervical joint radiofrequency neurotomy rates increased more sharply, at about 8.9% a year [7]. This reflects a broader trend toward minimally invasive, interventional pain management techniques for chronic neck pain, even as the clinical efficacy, patient selection, and long-term benefits remain subject to ongoing debate [8].

Despite their growing popularity, every aspect of these interventions is contentious. In response, the American Academy of Pain Medicine and the American Society of Regional Anesthesia and Pain Medicine formed the Cervical Joint Working Group in 2020 to create official guidelines. This represented 18 stakeholder organizations that nominated panelists who collaborated to create and refine clinical questions through a structured, consensus-based process known as the modified Delphi method. The group ultimately selected 20 basic questions related to the safety, effectiveness, and appropriate use of cervical joint interventions. These addressed subjects such as the status of clinical assessment and imaging, need for conservative treatment prior to injections, diagnostic and therapeutic value of different blocks, technical performance of radiofrequency neurotomy, and cut-off points for repeat interventions. Of these, 17 questions achieved full consensus by the committee members, with 14 out of 15 voting organizations supporting the guidelines [8]. This degree of concordance highlighted a firm professional consensus on important procedural standards and best practices.

The final guidelines concluded that cervical medial branch RFA can be beneficial for properly selected patients, with the procedure preceded by medial branch blocks (MBBs) with greater predictive value than intra-articular (IA) injections. More restrictive selection criteria would enhance outcomes, but at the expense of potentially increased false-negative rates and thus overall success rate. The panel explained that clinical practice and clinical trials could necessitate different standards and suggested flexibility in guideline application based on setting and purpose. There are no specific symptoms or physical examination findings that will reliably predict a positive response to facet joint blocks in chronic neck pain [9]. Whiplash history and tenderness over paraspinal muscles over the facet joints, on the other hand, are predictive of an increased likelihood of a positive response [10]. Radicular signs can be predictive of a negative diagnostic cervical MBBs response. Psychological variables do not seem to be involved in whether patients respond to these treatments. Target selection for blocks needs to be determined on a clinical presentation, including tenderness on palpation and pain referral patterns, although this is a low degree of certainty recommendation. Data currently available are not adequate to conclude that radiological imaging modalities properly balance the potential benefits and risks of diagnosing cervical facetogenic pain or predicting the success of cervical facet blocks or RFA [11]. MBBs are preferentially utilized over IA injections in the selection of the most appropriate candidates for RFA because they have been shown to be more diagnostically sensitive and predictive of a successful outcome with treatment. While MBBs are primarily utilized to ascertain whether or not the facet joint is painful, IA injections are therapeutically beneficial in certain circumstances, especially when there are no MBBs available. However, IA injections are less specific and sensitive to MBBs. Therefore, IA injections are usually reserved for specific clinical scenarios and not for routine diagnostic purposes before RFA [12].

Given the generally favorable course of acute neck pain, a six-week trial of conservative management is recommended before proceeding with prognostic cervical facet blocks, to avoid unnecessary invasive procedures and reduce healthcare costs. While conservative therapy may eliminate the need for further intervention, it does not exclude the use of blocks in patients who do not respond [13]. Fluoroscopy is the best practice for cervical MBB, with ultrasound (US) being provided by the providers with appropriate skill sets as an option where minimizing radiation is important. For IA injections, fluoroscopy should take the place of CT in favor of lesser exposure to radiation, though this is graded with less certainty. Similarly, for cervical medial branch RFA, fluoroscopy is chosen over CT for the same reason, with high certainty for imaging and moderate certainty for preferring fluoroscopy. While CT-fluoroscopy reduces radiation relative to CT, it is not yet widely available and carries added costs and radiation burden [14]. It is recommended that the amount of cervical MBB injectate be ≤0.3 mL, but it can be used with a slight increase in volume if the contrast does not reach the normal innervation patterns. For cervical IA facet joint injections, the contrast volume should not exceed 1 mL to reduce the risk of capsular rupture or unintended spread and hence increase block specificity [15].

There is a recommendation to use a near-parallel (posterior or slightly posterior oblique) approach to cervical medial branch RFA in all cervical spinal segments and in those with a history of previous anterior cervical surgery. The recommendation does not extend to other technologies, e.g., cooled radiofrequency (RF). In the case of prior surgery on the articular pillars, a variation technique such as an obliquely more posterior or lateral approach on the basis of sophisticated imaging may be required, possibly with multiple lesions [16]. Sensory stimulation should be applied, particularly when performing single lesions or when going in on the C2-3 facet, but the evidence is poor. For multiple lesion operations, the efficacy of sensory stimulation remains uncertain. Motor stimulation can enhance the efficacy and safety of the cervical medial branch RFA [17]. To maximize the probability of successful targeting of small cervical medial branches, the production of larger lesions can be beneficial, but anatomical limitations and the risk of damaging nearby vascular or neural structures must be considered with great care. When optimizing outcomes, creating a larger lesion may be preferable, whereas in situations where minimizing the risk of complications is the priority, using a smaller-gauge needle or a shorter active tip may be more appropriate, especially when treating the third occipital nerve (TON) [18]. But further safety investigations need to be undertaken before extensive application of newer systems like bipolar or cooled RF systems; the recommendations have low-to-moderate certainty. Table 3 provides a concise summary of the consensus practice guidelines on interventions for cervical spine (facet) joint pain.

**Table 3:** Summary of recommendations. AA, atlanto-axial; AO, atlanto-occipital; DSA, digital subtraction angiography; IA, intra-arterial; MBB, medial branch block; NSAID, non-steroidal anti-inflammatory drug; RFA, radiofrequency ablation; TON, third occipital nerve; US: ultrasound.

Question	Recommendation	Grade	Level of certainty
Use of history and physical examination to identify painful AO or AA joints or to select patients for injections	Cannot reliably identify painful joints, but can guide injections	Grade C	Low
Use of history and physical examination to identify painful cervical facet joints and select patients for blocks	No reliable signs to predict response	Grade C	Low
Correlation between radiological findings and prognostic block or RFA outcomes	Evidence insufficient; consider imaging for planning	Grade I; Grade C	N/A; Low
Conservative treatment requirements before cervical facet blocks	Should be used before blocks; 6-week trial preferred	Grade B; Grade C; Grade I	Moderate; Low; N/A
Image guidance for cervical facet blocks and RFA	Fluoroscopy/US for MBB (A); fluoroscopy for IA (C); fluoroscopy for RFA (A)	Grade A; Grade C; Grade A/B	Moderate; Low; High/Moderate
Optimal technique for AO and AA joint injections and risk mitigation	Use imaging; posterior approach; limited steroid dose	Grade C; Grade B; Grade C	Low; Moderate; Low
Approach for cervical MBB	Lateral approach for TON, C3–C7; posterior for C8; small needles	Grade I	N/A
Volumes for cervical MBB and IA injections	<0.3 mL for MBB, ≤1 mL for IA	Grade C (both)	Low
Therapeutic value of cervical MBB and IA injections	IA is not routinely used; avoid steroids in MBB	Grade C; Grade D	Low–Moderate; Moderate
Performing bilateral cervical MBB and RFA, and level limits	Bilateral MBB appropriate; consider single rather than bilateral RFA; ≤2 levels/session	Grade C	Low
Diagnostic and prognostic utility of cervical injections	MBB predictive; IA has a high failure rate; AO/AA may help	Grade C	Low–Moderate
Use of sedation	Not routinely for diagnostics	Grade B	Moderate
Cut-off for designating an MBB as positive and the use of non-pain measures	≥50% relief; non-pain measures supplementary only	Grade C; Grade B	Low–Moderate; Moderate
Number of blocks before RFA	Single block recommended unless exceptions apply	Grade B	Low–Moderate
Orientation of electrodes for RFA	Near-parallel preferred; modified approach for prior surgery	Grade B; Grade C	Low–Moderate; Low
Sensory and motor stimulation before RFA	Sensory for single lesions; motor for safety/efficacy	Grade C; Grade B	Low; Low–Moderate
Evidence for larger lesions	May improve results and duration	Grade C	Low–Moderate
Risk mitigation	Real-time fluoroscopy; manage anticoagulants carefully; consider device compatibility	Grade B/C/I	Moderate/Low/N/A
Repeating RFA	Repeat if ≥3 months of relief; no need to repeat MBBs if pain recurs similarly	Grade B; Grade C	Moderate; Low
Differences between clinical trials and practice	Patient selection and performance may differ	–	–

Lumbar facet joint interventions

Over the past two decades, lumbar facet blocks and RFAs have gained wide acceptance for the treatment of low back pain (LBP). The accumulation of evidence from ongoing studies continues to put the indications, effectiveness, and overall clinical role of these interventions into sharper focus. In an effort to circumvent this problem, the American Society of Regional Anesthesia and Pain Medicine initiated a guideline development process among a number of pain societies and U.S. Department of Veterans Affairs and Department of Defense members. A steering committee set and strengthened 17 key questions using a modified Delphi process, aiming for expert consensus. The questions covered topics such as the application of physical examination and imaging for selection of patients, conservative regimens before blocks, whether to use MBB or IA injections for diagnosis and prognosis, and optimal procedural technique with sedation, amount of injectate, and placement of electrodes. The result was complete consensus between committee members on all questions except for one of the participating societies, which had two differences (number of blocks and the cut-off before a positive block before RFA), but still supported the overall guideline [19]. Final recommendations emphasized that lumbar medial branch RFA may be beneficial in properly selected patients, with MBBs proving to be more predictive than IA injections. The recommendations note that stricter selection criteria may enhance outcomes but could create more false negatives. Interestingly, the approach in clinical trials may diverge from everyday practice, with trials often having to run on tighter criteria in a bid to make research goals possible. The recommendations overall tend to balance efficacy, safety, and feasibility in research and clinical settings.

Specific physical examination findings or history characteristics are unable to reliably predict which patients with chronic axial LBP will respond to facet joint blocks [20]. Non-midline pain and facet joint point tenderness may be weakly associated with a positive response, but radicular symptoms are likely to predict failure. When selecting block targets, clinicians need to consider the overall clinical presentation, including radiological findings (if any), fluoroscopically guided tenderness, and characteristic pain referral patterns. Imaging has also been used to establish radiological evidence of painful lumbar facet joints, and IA injections or MBBs have been the conventional gold standard employed for verification of pain origins. Among the imaging modalities available, single-photon emission computed tomography (SPECT) has been the most thoroughly studied for facet joint detection that is potentially painful. But SPECT involves intravenous administration of a gamma-emitting radionuclide and subjects patients to higher radiation doses than do standard X-rays [21].

Existing clinical practice guidelines (CPGs) for LBP advocate patient-centered care that includes screening for psychosocial problems, education on back pain, and motivation to exercise and physical therapy. Thus, the timing and length of LBP treatment are highly individualized. When primary care and acute care physicians adhere to guidelines, patients referred to pain specialists will most likely have already undergone several conservative forms of treatment [22]. A three-month trial of conservative management is recommended before trying facet joint procedures. These include medications like nonsteroidal anti-inflammatory drugs or antidepressants, physical modalities like exercise and application of heat or cold, and massage, and integrative care like acupuncture or spinal manipulation when appropriate. Other supportive therapies, such as maximization of nutrition, weight management, and sleep hygiene, may also be beneficial.

Image guidance has become a permanent part of performing spinal procedures for pain management. Fluoroscopy and, to a lesser degree, CT guidance are most commonly applied for MBB and IA facet joint injections. Imaging makes it possible to use proper needle placement, which ensures the minimum amount of anesthetic is injected, thereby reducing spread to contiguous structures, which could lead to false-positive test results. Image guidance also increases safety by direct visualization of bony structures of the neuraxis, thus avoiding structures nearby like pleura, neural foramina, and blood supply [23]. Fluoroscopy is preferred over CT for lumbar MBBs due to its lower cost, faster procedure time, and reduced radiation exposure, though ultrasound may be appropriate for patients where radiation poses risks or when imaging resources are limited. For IA injections, CT is recommended for greater accuracy, but fluoroscopy with contrast may be acceptable in select patients, such as those who are thin and have minimal joint narrowing. For lumbar medial branch RFA, fluoroscopy is recommended since CT offers no added benefit and exposes patients to higher radiation levels.

The terms diagnostic, prognostic, and predictive are often used interchangeably in the chronic LBP literature, but they have distinct meanings. Diagnosis involves identifying a disease or condition based on its signs and symptoms, while prognosis refers to predicting the likely course or outcome of that condition, including potential treatment effects. In contrast, predictive describes how likely a specific therapeutic intervention is to produce a particular effect, and although these concepts may overlap, they represent different aspects of clinical evaluation [24]. IA facet joint injections are diagnostic injections in the diagnosis of facet-mediated pain, but are less predictive of response to medial branch RFA and have a higher technical failure rate. MBBs, while useful, are encumbered by lumbar facet joint variability of innervation. Local anesthetic injections of IA and MBBs compared with saline controls are more predictive of the outcome of RFA.

Previous reports have suggested that lumbar MBBs and IA injections are similarly effective in identifying painful facet joints and selecting appropriate patients for RFA [25]. IA facet joint injections qualify as diagnostic tools for facet-mediated pain but are less predictive of response to medial branch RFA and have a higher technical failure rate. MBBs, despite limitations from variable lumbar facet joint innervation, along with IA injections using local anesthetic, offer better predictive value for RFA outcomes compared to saline controls. MBBs are the primary prognostic screening test preceding lumbar facet RFA. IA corticosteroid injections may be therapeutically beneficial and may be utilized as prognostic blocks in certain populations, for instance, young, athletic patients, wherein muscle denervation will cause atrophy with effect on function, or in those at risk of complications from RFA, for instance, in the presence of implantable cardioverter-defibrillators. Though RFA can be used in patients with implantable devices, there have been limited cases of defibrillator malfunction despite precautions. Younger patients with acute inflammatory facetogenic pain can be sensitive to IA steroids and also tend to have a lower technical failure rate since they have fewer osteoarthritic changes.

Sedation should not be routinely used for diagnostic or prognostic facet injections unless there are clear indications [26]. If sedation is necessary, patients should be informed about the increased risk of false-positive results, and the lowest effective doses of short-acting sedatives, preferably without opioids, should be administered. The facet joint is a true synovial joint and holds 1.0-2.0 mL of fluid, and too much as well as too little injectate can compromise the validity of diagnostic blocks [27]. High volumes have the risk of capsular rupture and spread to other sources of pain and reduced specificity, and low volumes carry the risk of incomplete anesthesia and false negatives. Due to the brief duration and limited long-term efficacy of IA blocks, there are few correlations with injectate volume and relief of pain. Less than 0.5 mL total volume should be employed for lumbar MBBs and ≤1.5 mL for lumbar IA facet joint injections to limit spread and capsular damage.

A reduction in pain of ≥50% is considered a positive block, though further studies are needed to determine if lower thresholds might be more effective. While patients experiencing 80% or more relief tend to respond better to RFA [28], many with 50-79% relief also benefit, so the committee recommends using the 50% cut-off to maximize patient access to care. Secondary factors such as medication use, activity levels, and patient satisfaction should also influence the decision to proceed with RFA, ensuring that pain relief is genuine and not due to other factors like sedation. Future clinical prediction models may refine these cut-off values for different scenarios. The committee recommends using a single MBB before RFA. Although dual blocks show moderate evidence of higher success rates, a zero-block approach yields the highest number of positive RFA responses [29]. Therefore, accepting a single MBB is a practical compromise, with some data indicating better outcomes in patients reporting greater relief from that block. The committee supports tailoring treatment based on individual patient factors and clinical goals, reflecting a personalized medicine approach. Due to the small size of lumbar medial branches and dorsal rami, creating larger lesions during thermal RFA may improve the chances of targeting these nerves. However, caution is needed to avoid damaging nearby non-targeted structures [30]. There is low-certainty evidence supporting the use of larger lesions to better capture nerves, but insufficient evidence to confirm if larger lesions prolong pain relief.

The committee recommends repeating lumbar medial branch RFA when pain recurs after at least three months of relief, preferably six months for those undergoing multiple procedures, and advises limiting the procedure to no more than twice per year [31]. Although success rates may decline over time, this approach balances effectiveness and safety. Routine repeat prognostic blocks are not recommended if the returning pain resembles the original pain, but they may be considered when the pain’s nature or source is uncertain. The following table (Table 4) provides a summary of recommendations to manage lumbar facet joint pain based on the final guidelines by the steering committee.

**Table 4:** Summary of recommendation for lumbar facet pain. IA, intra-articular; LA, local anesthetic; MBB, medial branch block; RF, radiofrequency; RFA, radiofrequency ablation; SPECT, single photon emission computed tomography.

Topic	Summary	Recommendation grade/certainty
Value of history & physical exam	No reliable signs predict lumbar facet block response; paraspinal tenderness may predict positive, radicular symptoms negative. Target levels based on clinical presentation.	Grade C, Low certainty
Imaging correlation & necessity	Moderate evidence for SPECT before MBB; weak evidence for SPECT, MRI, CT, scintigraphy before IA or MBB.	Grade C (SPECT/MBB), Moderate; Grade D (others), Low
Conservative treatment before blocks	A 3-month trial of conservative therapies is recommended before facet interventions.	Grade C, Low certainty
Imaging guidance for blocks & RFA	Fluoroscopy or CT for MBB; CT preferred for IA, fluoroscopy acceptable. Fluoroscopy recommended for RFA.	Grade C (blocks), Low; Grade B (RFA), Low
Diagnostic/prognostic value of blocks	IA is more diagnostic but has a high failure rate; both IA and MBB are better than placebo before RFA.	Grade B, Low certainty
MBB vs. IA before RFA	MBB preferred before RFA; IA may be used in select patients (e.g., inflammatory pain and RFA risk).	Grade C, Moderate certainty
Effect of sedation	Sedation should not be routine unless individually indicated.	Grade B, Low–Moderate certainty
Ideal volume for blocks	<0.5 mL for MBB, <1.5 mL for IA to avoid spread and rupture.	Grade C, Low certainty
Therapeutic value of MBB & IA	Routine use not recommended, but may help in select scenarios (e.g., prolonged relief and RFA contraindications).	Grade D, Moderate certainty
Cut-off for positive block	A higher threshold may improve RFA outcomes. RFA with at least 50-80% pain relief had a 75% success rate for 1 year, while RFA with >80% pain relief had a 93% success rate for 1 year. Activity measures may also guide decisions.	Grade B, Moderate certainty
Number of blocks before RFA	Recommend single block; multiple blocks increase specificity and increase false negative rates, thus resulting in decreased access to care.	Grade C, Low–Moderate certainty
Evidence for large RF lesions	Indirect evidence supports larger lesions (e.g., larger electrodes and higher temperatures) for capturing nerves. Limited evidence for longer relief.	Grade C (capture), Low; Grade I (duration), Low
Electrode orientation	Recommend near-parallel orientation to the nerve.	Grade B, Low certainty
Sensory & motor stimulation	Sensory stimulation is advised with single lesions; inconclusive with multiple lesions. Motor stimulation may aid safety and effectiveness.	Grade C (sensory), Low; Grade I (multi-lesion); Grade B (motor), Low
Complication mitigation	Use aspiration and real-time contrast to prevent vascular uptake. Continue anticoagulants unless clear indication. Steroid post-RFA may prevent neuritis. Sensorimotor testing can reduce nerve injury.	Grade B–C, Mostly Low certainty
	Discuss muscle/disc degeneration risks with patients; use physical therapy pre/post RFA to mitigate.	Grade C, Low certainty
	Consider device interference with implanted electronics. Use bipolar settings and proper grounding. Avoid excessive sedation.	Grade C, Low certainty
	Burns are rare but possible; minimize risk by equipment checks and proper grounding pad placement.	Grade B, Moderate–High certainty
	Spine surgery lowers RFA success; use multiple fluoroscopic views and avoid hardware contact.	Grade C, Low certainty
Clinical trials vs. practice	Trial protocols may differ from clinical practice in patient selection, block number, contrast use, and stimulation.	Grade A, Moderate certainty
Repeating RFA	Can be repeated up to 2x/year if ≥3 months of relief; success decreases over time but stays >50%.	Grade B, Moderate certainty
Repeating prognostic blocks	Not routinely needed if pain recurs in the expected pattern/timeframe.	Grade C, Low certainty

Sacroiliac joint disorders

The sacroiliac joint (SIJ) is a common source of pain, arising either as a primary condition or secondary to factors such as gait abnormalities, spinal disorders, or specific risk factors. Despite its high prevalence, SI joint dysfunction is often underdiagnosed and may be inadequately managed or treated with unnecessarily invasive procedures. Accurate diagnosis and effective treatment of SI joint-mediated pain remain challenging due to overlapping clinical presentations, variable diagnostic criteria, and evolving interventional techniques. Despite the availability of multiple treatment modalities such as diagnostic injections, therapeutic corticosteroid injections, RFA, and minimally invasive SI joint fusion, practice patterns among providers still remain significantly diverse.

To address this heterogeneity and promote evidence-based, standardized care, the ASPN convened an expert multidisciplinary panel to develop the best practice guideline for the treatment of sacroiliac disorders [32]. Of course, a brief synopsis of the initial work is required, synthesizing its primary diagnostic and interventional suggestions into practicable advice for effective utilization in the clinic. The current synopsis attempts to discern the most important recommendations of the ASPN consensus to support informed, uniform, and effective treatment of SI joint-related lower back pain.

Diagnosis of SI Joint Dysfunction

A thorough patient history must be obtained to ascertain symptoms consistent with SIJ dysfunction (SID) and assess for risk factors that increase the likelihood that this disorder is the etiology of lower back pain. Physical examination is most valuable in diagnosis, one of the more sensitive signs being the Fortin finger test, which is positive if one elicits pain referred within 1 cm of the posterior superior iliac spine. Laslett et al. showed that the presence of three or more of five particular provocative tests is very indicative of SID diagnosis [33]. The tests are SI joint distraction, SI joint compression, Gaenslen's maneuver, Patrick's test, and the thigh thrust test. All these maneuvers are considered positive when they cause the patient's characteristic pain. Table 5 provides a summary of recommendations to facilitate the diagnosis of SIJ dysfunction.

**Table 5:** Diagnosis of SIJ dysfunction. SIJ, sacroiliac joint; CT, computed tomography; MRI, magnetic resonance imaging.

Diagnostic method	Specific criteria/maneuvers
Provocative maneuvers	At least three out of the following five maneuvers must be positive: • SIJ distraction • SIJ compression • Gaenslen’s maneuver • Patrick’s test • thigh thrust
Intra-articular SIJ injections	>50% pain relief with a local anesthetic intra-articular SIJ injection
Imaging	The absence of other sources of pathology on imaging (CT/MRI)

Conservative Care of SI Joint Disorders

Conservative treatment of SIJ dysfunction aims to reduce pain, normalize hip and pelvic mechanics, and improve function and activity levels. Physical therapy, anti-inflammatory medications, and external supports, such as pelvic belts, form initial treatment, with an emphasis on optimizing these endeavors before proceeding to interventional or surgical techniques. Acute SIJ pain will typically resolve with conservative management, beginning with initial activity modification for a short duration, followed by targeted physical therapy to restore mobility, strengthen, and correct muscle or postural imbalances [34]. Sacroiliac belts can be used to stabilize the pelvis and improve function, especially when combined with physical therapy, though evidence for pain reduction over the long term is limited. Medications such as non-steroidal anti-inflammatory drugs (NSAIDs) are first-line for pain, with muscle relaxants and topical medications on an as-needed basis. For the chronic phase, patients are recommended to maintain a home exercise program and use medication on an as-needed basis, reserving invasive procedures for times when pain is ongoing. Table 6 provides recommendations on conservative care of SIJ disorders.

**Table 6:** Conservative care of sacroiliac joint disorders. NSAID, non-steroidal anti-inflammatory drug.

Phase of care	Recommendations
Acute (1–3 days)	Avoid triggering activities and use medications such as NSAIDs.
Subacute (3 days - 8 weeks)	Focus on increasing activity, mobility, and strengthening. Patients should begin a physical therapy program with a focus on stretching, normalizing posture, and improving body mechanics. Medications like NSAIDs may continue to be used.
Chronic/maintenance phase (>8 weeks)	This phase includes interventional pain management options and a consistent home exercise program. Medications like NSAIDs may be used as needed.

SI Joint Injections

SIJ injections are both diagnostic and therapeutic and typically are performed under image guidance, most commonly under fluoroscopy, with CT and ultrasound also being used occasionally [35]. The absolute contraindications are local malignancy and infection, while the relative contraindications include coagulopathy, pregnancy (based on the type of imaging), and systemic infection. Diagnosis is established by a combined series of clinical tests, three or more positive provocative maneuvers, and proof of >50% relief of pain following image-guided IA injection. CT delivers maximum accuracy for diagnostic injections, but is less utilized as fluoroscopy, though not as accurate, is more widely available and more cost-effective, and has therefore become the norm, with ultrasound providing a radiation-free alternative. Complications are usually transient and mild and include pain exacerbation or minor nerve irritation, though rare cases of septic arthritis may occur in high-risk patients [36]. Table 7 summarizes best practice recommendations for SIJ injections.

**Table 7:** Best practice recommendations for sacroiliac joint injections. IA, intra-articular; CT, computed tomography; SIJ, sacroiliac joint; US, ultrasound; PRP, platelet-rich plasma; PRF, platelet-rich fibrin.

Recommendation	Grade of recommendation
Fluoroscopic and CT guidance should be used for SIJ injection, given the safety and increased duration of relief.	B
US guidance can be considered for SIJ injections where radiation exposure may be problematic.	B
IA steroid injections may offer benefit compared to conservative therapy for sacroiliitis.	A
PRP and PRF IA injections may offer benefits compared to conservative therapy for sacroiliitis.	B
Diagnostic SIJ IA injections combined with a physical exam are effective in determining and confirming sacroiliitis when utilized jointly.	A

Neuroablative Procedures in SI Joint Dysfunction

Neuroablative treatments, particularly RFA, are considered for SIJ pain after conservative measures and positive diagnostic blocks. The most common approach targets the lateral branches of the S1-S3 dorsal rami and the medial branches of the L5 (±L4) dorsal rami, while other modalities like cryoablation or chemical neurolysis show limited benefit and higher risks [37]. Diagnostic confirmation is typically achieved using multi-site, multi-depth lateral branch blocks under fluoroscopy or ultrasound, with ≥50% pain relief considered a sufficient threshold to proceed with RFA [38]. Studies show significant pain relief and functional improvement following RFA in appropriately selected patients, though dual diagnostic blocks are preferred to minimize false positives. Contraindications include sacral fracture, malignancy, infection, and coagulopathy, and the technique’s precision is crucial to effectively target the extra-articular pain sources associated with SIJ dysfunction [39]. Table 8 provides the best practice recommendations on SI joint RFA.

**Table 8:** Best practice recommendations for sacroiliac joint radiofrequency ablation. SIJ, sacroiliac joint; RFA, radiofrequency ablation.

Recommendation	Grade of recommendation
SIJ RFA may be considered for the treatment of sacroiliitis that is refractory to conventional conservative care.	B
There are no comparative studies; thus, there is no recommended preferred modality of SIJ RFA for sacroiliitis.	I
SIJ RFA seems to provide longer-lasting relief compared to SIJ steroid injections for the treatment of sacroiliitis.	C

Minimally Invasive Sacroiliac Joint Fusion

Regenerative medicine for SIJ dysfunction remains an emerging treatment supported by limited evidence and should be considered only when established therapies fail or are contraindicated. Minimally invasive surgical options for SIJ fixation include posterior, oblique, and lateral approaches, each with specific diagnostic and procedural criteria such as confirmed SIJ dysfunction through history, physical exam, and diagnostic injections. Posterior fusion using allografts or titanium implants has shown significant pain reduction and functional improvement with minimal complications [40]. The posterior oblique approach involves placement of one to three implants across the SIJ, providing stabilization through a less invasive route. The lateral minimally invasive approach is currently the most common and well-studied technique, demonstrating marked improvements in pain, disability, and quality of life while reducing opioid use and posing fewer risks compared to traditional open fusion surgeries [41]. Table 9 provides the summary of ASPN best practice statements on minimally invasive sacroiliac fusion [32].

**Table 9:** ASPN best practice statements for minimally invasive sacroiliac fusion. ASPN, American Society of Pain and Neuroscience; SI, sacroiliac; SIJ, sacroiliac joint; VAS, visual analog scale; NRS, numeric rating scale; ODI, Oswestry Disability Index.

Recommendation	Inclusions & exclusions	Grade of recommendation
Minimally invasive posterior SI stabilization with allograft is considered medically necessary when the appropriate clinical criteria have been met.	Inclusions: 1. Failure of conservative measures (e.g., physical therapy and injections). 2. Pain >6 months interfering with function (VAS/NRS ≥5, ODI ≥30). 3. Failure of at least one therapeutic SIJ injection (>50% pain relief for <3 months). 4. Predominant pain pattern consistent with SIJ pathology. 5. Positive response from at least three validated maneuvers. 6. Positive Fortin finger test. 7. Diagnostic imaging (CT or MRI) excludes destructive lesions. 8. Diagnostic confirmation via image-guided intra-articular SIJ injection with ≥50% pain relief. Exclusions: 1. Infection or fracture. 2. Tumor. 3. Acute traumatic instability.	A
Minimally invasive SI fusion with lateral transfixing devices is considered medically necessary when the appropriate clinical criteria have been met (as above).	Same as above.	A
Minimally invasive SI fusion implants should be used according to the FDA labeling.	N/A	A
The use of implants composed of human cell and tissue products for sacroiliac fusion is considered medically necessary only if the FDA regulations and registration are followed.	N/A	A
ASPN supports the utilization of devices with published, peer-reviewed, multi-center, prospective evidence of at least 6 months to assess efficacy and safety.	N/A	A
The current evidence is insufficient to determine the medical necessity of emerging techniques such as posterior-transfixing and hybrid approaches.	N/A	I

Discussion

This collection of international consensus statements regarding cervical and lumbar facet interventions and SI joint disorders identifies broad consensus regarding diagnostic criteria, image guidance, and measured escalation of care, with areas of sparse evidence marked along the way. Across sources, controlled diagnostic blocks with prespecified levels, and a stringent technique for RFA, are echoed as necessary principles. For SI pathology, uniform provocative testing combined with image-guided IA diagnostic blocks serves as the anchor for decision-making, with increasing to injections, RFA, and, in refractory scenarios under stringent criteria, minimally invasive fusion [32].

There is strong agreement that prognostic MBBs better predict success with facet RFA than IA injections when planning RFA, but IA injections are a good option for short-term relief or if RFA cannot be done [42]. Real-time imaging guidance is taken to be standard: fluoroscopy as the preferred option, and ultrasound as acceptable in some cervical applications [43]. CT is generally held for difficult anatomy or guided IA procedures. They all consider ≥50% pain relief clinically significant for block success, with some recommending higher levels to be more sensitive to improvement [44]. Technical guidelines for RFA stress near-parallel orientation of the probe, testing of sensory/motor function, small injectate volumes, and future repetition, dependent on the patients' benefit [19].

Considerable variation among guidelines exists. Single vs. dual prognostic blocks before RFA tips faster access and lower cost against reduced false positives, especially in cervical facet joint pain [14]. IA steroids for chronic facet pain are generally avoided with a modest duration, but still an acceptable option for non-RFA candidates [17]. In SI joint pain, IA steroids receive more support [45]. Operator expertise, anatomy, and equipment are all accommodated in modality choice: ultrasound reduces the dose of radiation, and CT provides specificity at a higher dose and cost [19]. Technique details such as lesion contour, overlap protocol, and stimulation parameters are more dictated by the skilled practitioner. Relative effectiveness of RFA modalities versus IA steroid and fusion in SI conditions is more in need of standardized head-to-head trials under standardized selection criteria [42]. Strengths of the review include conversion of voluminous advice into steps conforming to the clinic; weaknesses are dependence on methodological quality of source papers and dependence on expert agreement where grades of evidence are weaker.

## Conclusions

In conclusion, chronic back pain resulting from facet or SIJ disease remains an arduous and burdensome condition that calls for appropriate, evidence-based treatment. Although existing international and national guidelines represent a robust synthesis of expert views and emerging evidence, their technical complexity often renders them unworkable in day-to-day clinical practice. Creating an accessible, practice-based summary of these authoritative guidelines may bridge the gap between study and practice. Through providing concise, graded, and actionable recommendations, such a resource would empower clinicians to deliver consistent, high-quality, and value-based care, ultimately leading to better outcomes and quality of life in patients with chronic spinal pain.
